# Immune Characteristics of LYN in Tumor Microenvironment of Gliomas

**DOI:** 10.3389/fcell.2021.760929

**Published:** 2022-02-02

**Authors:** Chonghua Jiang, Hao Zhang, Wantao Wu, Zeyu Wang, Ziyu Dai, Liyang Zhang, Zhixiong Liu, Quan Cheng

**Affiliations:** ^1^ Department of Neurosurgery, Xiangya Hospital, Central South University, Changsha, China; ^2^ National Clinical Research Center for Geriatric Disorders, Xiangya Hospital, Central South University, Changsha, China; ^3^ Department of Oncology, Xiangya Hospital, Central South University, Changsha, China; ^4^ Department of Medicine, The University of Oklahoma Health Sciences Center, Oklahoma City, OK, United States; ^5^ Clinical Diagnosis and Therapeutic Center of Glioma, Xiangya Hospital, Central South University, Changsha, China

**Keywords:** glioma, lyn, microenvironment, immune suppression, prognostic marker, PD-L1

## Abstract

The prognosis of gliomas is poor and there are limited therapeutic approaches. Immunotherapy has become a promising treatment for gliomas. Here, we explored the expression pattern of Lck/yes-related protein tyrosine kinase (LYN) in gliomas and assessed its value as an immunotherapy biomarker. Transcriptional data was mined from two publicly available datasets, TCGA and CGGA, and used to investigate the correlation between LYN and clinical characteristics including patient prognosis, somatic mutation, and immune infiltrating features in gliomas. Besides, the correlation between LYN and classical immune checkpoint molecules was explored. Glioma samples obtained from the Xiangya Hospital cohort were used for immunohistochemistry staining. High expression level of LYN was observed in advanced gliomas and other cancer types, which predicted a worse prognosis. LYN stratified patients’ survival in the Xiangya cohort and was also significantly associated with infiltrating immune cell types and inflammatory activities in the tumor microenvironment. LYN was involved in tumor mutation, correlated with the regulation of oncogenic genes, and also showed a significant positive correlation with PD-L1. LYN can be a potential diagnostic marker and immunotherapy marker in gliomas.

## Introduction

Gliomas are the most malignant tumors. The latest world health organization (WHO) category defines grade 2 and grade 3 glioma as diffuse lower-grade glioma (LGG), and grade 4 gliomas as glioblastoma (GBM). Although the classical treatment options of surgery and adjuvant chemoradiotherapy are reported, the median overall survival rate of glioma patients is still less than 10 years (Zhang et al., 2019). Besides, it is noteworthy that, although patients with LGG enjoy a relatively favorable prognosis, most LGG eventually progress to GBM (Claus et al., 2015). The dismal outcome, tumor recurrence, and inevitable drug resistance reveal the urgent need to explore potential biomarkers involved in the tumorigenic mechanism of gliomas and develop potential therapeutic targets for treatment of glioma patients (Zhang et al., 2021a).

Gene markers are becoming increasingly attractive in tumor research, including predicting tumor progression and treatment efficiency, reducing the recurrence rate, and prolonging patients’ survival (Zhang et al., 2021b). Immune checkpoint molecules have been identified as effective markers in predicting the immunotherapeutic effect in various cancer types (Zhang et al., 2021c). Programmed death 1 (PD-1) and its ligand, programmed death-ligand 1 (PD-L1), have exhibited remarkable prognostic and therapeutic value (Billon et al., 2019; Dong et al., 2021). Previous studies with immune checkpoint inhibitors (ICIs) targeting PD1 and PD-L1 have demonstrated durable clinical responses and prolonged survival in solid tumors (Ribas and Wolchok, 2018). Notably, the reliability and efficacy of PD-1 and PD-L1 as immunosuppressive biomarkers have been explored and confirmed (Dunn-Pirio and Vlahovic, 2017; Dong et al., 2021). Given the positive rate of PD-L1 in the majority of gliomas, multiple clinical trials with PD-1/PD-L1 inhibitors in gliomas are ongoing.

As a member of protein tyrosine kinases, LYN critically regulates essential cellular processes including cell growth and cell differentiation. LYN is found predominantly in myeloid cells, B lymphocytes, and cell types outside of the hematopoietic compartment (Uhlén et al., 2015). Studies have demonstrated that LYN regulates positive and negative pathways in B cell-mediated immunity (Brodie et al., 2018). Moreover, LYN profoundly affects the innate immune responses by controlling the activation of dendritic and NK cells (Krebs et al., 2012).

In this study, we speculated that LYN may be another promising biomarker for immunotherapy in glioma patients. Using transcriptomic data from two datasets, we investigated the expression level, clinical features, and functional annotation of LYN. Notably, we found the co-expression pattern of LYN and PD-L1 in glioma microenvironment. LYN could also significantly predict the anti-PD-1 and anti-CTLA-4 immunotherapy responses.

## Results

### LYN Expression Correlates With Malignant Phenotypes in Gliomas

**FIGURE 1:**
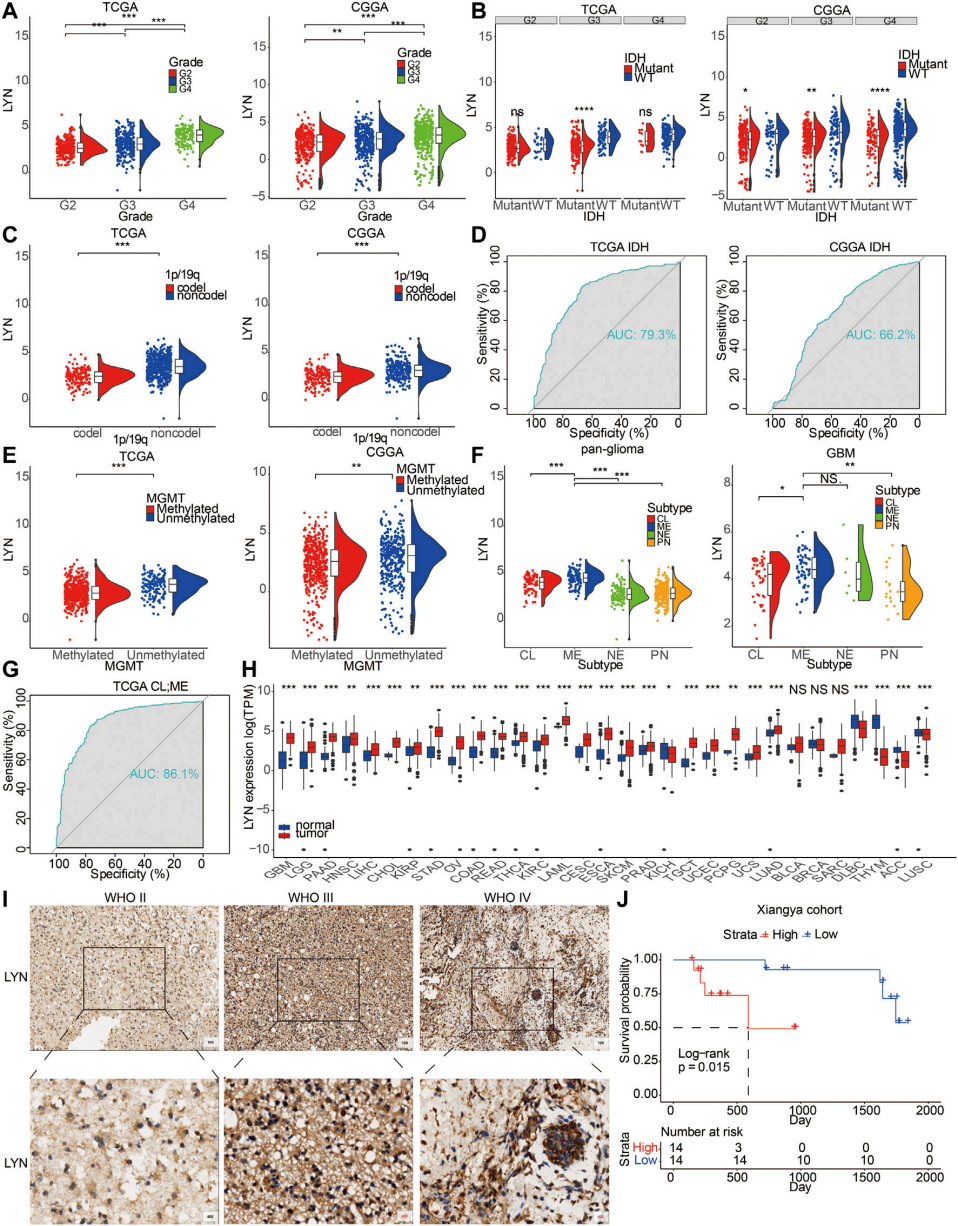
LYN expression correlated with diverse pahological characteristics **(A)**. LYN expression in different WHO grades in TCGA and CGGA **(B)**. LYN expression in different IDH state in TCGA and CGGA **(C)**. LYN expression in different 1p/19q status from TCGA and CGGA dataset **(D)**. ROC curves showed LYN as a predictor of IDH mutation **(E)**. LYN expression in different MGMT promotor status from TCGA and CGGA dataset **(F)**. LYN expression in GBM samples and glioma samples based on molecular subtypes in TCGA **(G)**. ROC curves of LYN in predicting classical and mesenchymal subtype glioma **(H)**. LYN expression in different cancers from TCGA dataset. *p < 0.05, **p < 0.01, ***p < 0.001 **(I)**. Immunohistochemistry staining results of LYN in Xiangya cohort **(J)**. Kaplan—Meier curve of overall survival in Xiangya cohort based on the staining intensity of LYN. *p*-value was obtained from the log-rank test.

LYN expression in GBM samples was higher than that in LGG samples (Figure 1A). In this study, LYN expression was observed in IDH wild-type gliomas (Figure 1B). It should be noted that LYN expression in TCGA was only significantly different in grade 3, while there were differences in all three grades in CGGA. The seemingly contradictory result could be explained by the insufficient samples in TCGA (672 samples) compared with CGGA (1,018 samples). Besides, the median value of LYN in WT group was generally higher than that in mutant group in all three grades in TCGA. ROC curve proved the predictive value of LYN expression in glioma patients with IDH mutation or IDH wildtype in TCGA and CGGA (Figure 1D). Furthermore, LYN showed high expression in histopathologically malignant glioma samples (Supplementary Figure S1B) and 1p/19q non-codeletion glioma samples (Figure 1C). LYN expression was more enriched in unmethylated glioma samples (Figure 1E). As shown in Figure 1F, LYN expression was more enriched in the classical (CL) and mesenchymal (ME) subtypes compared with proneural (PN) and neural (NE) subtypes in TCGA (Figure 1F). Meanwhile, the ROC curve indicated that LYN expression effectively predicted CL and ME subtypes (Figure 1G).

Patients with recurrent gliomas were detected with higher level of LYN expression compared with patients with primary gliomas (Supplementary Figure S1E). LYN showed higher expression in glioma patients with progressive disease than in glioma patients with complete remission (Supplementary Figure S1F). Additionally, LYN expression was higher in contrast-enhanced (CE) regions than in non-contrast-enhanced (NCE) and normal tissues (NT) (Supplementary Figure S1C). Besides, based on the tumor anatomic structure of GBM, LYN expression was more enriched in hyperplastic blood vessels (Supplementary Figure S1D). LYN protein level was also found to be upregulated with increase of WHO grades based on the IHC staining results of glioma samples (*n* = 40) from the Xiangya hospital cohort (Figure 1I). The expression level of LYN also stratified glioma patients’ survival in the Xiangya cohort (Figure 1J). The clinical information of the Xiangya cohort was provided in Supplementary Table S1.

Further, LYN expression levels in HMC-1 (mast cell), HEL (human erythroleukemia cells), U-698 (human B cell lymphoma), HDLM-2 (human Hodgkin lymphoma), and Karpas-707 (human myeloma) cell lineages were relatively high based on Cancer Cell Line Encyclopedia (CCLE) (Supplementary Figure S1A). Likewise, LYN was highly expressed in various cancer types (Figure 1H).

### LYN Expression Predicts Worse Survival of Glioma Patients

**FIGURE 2:**
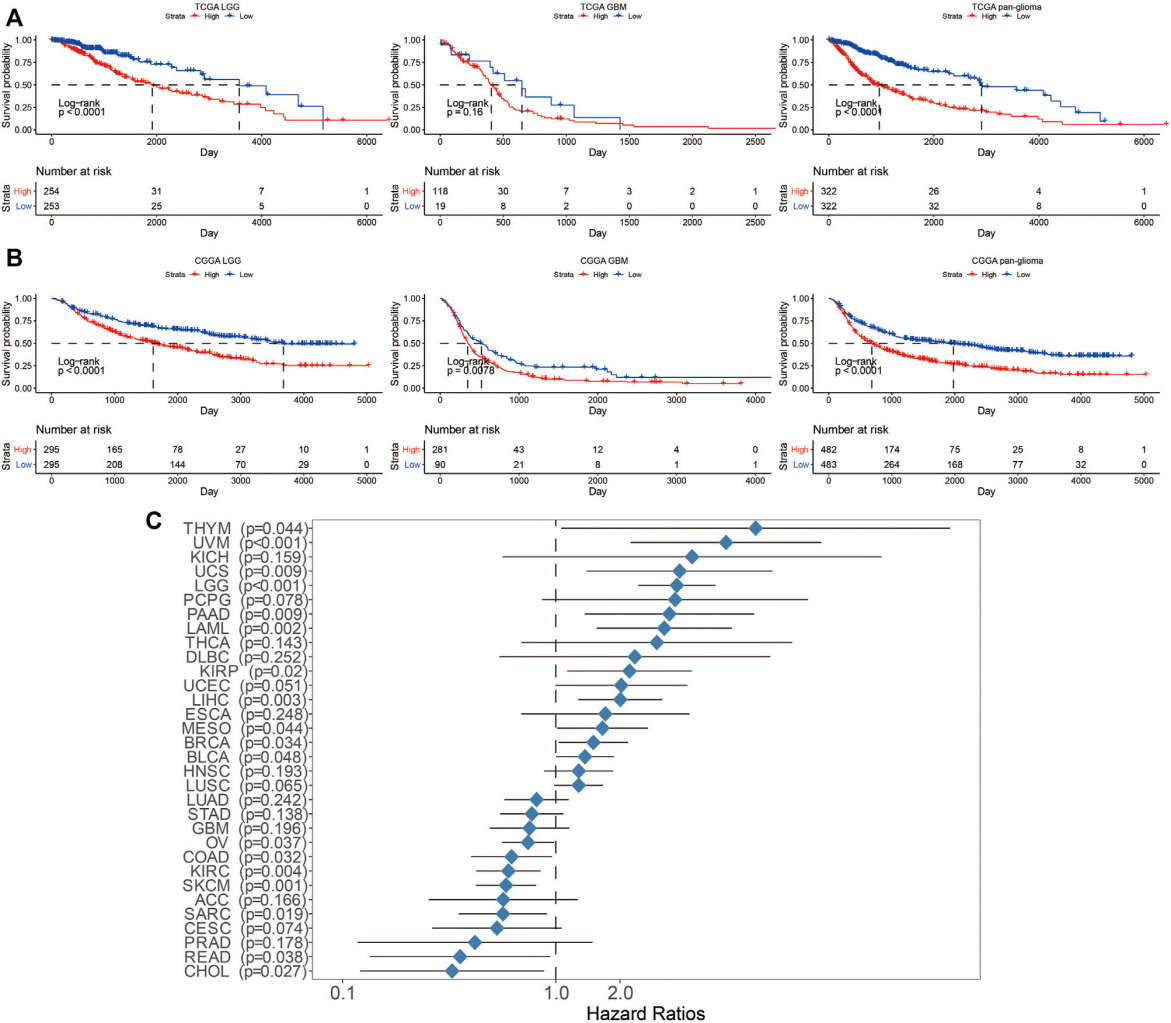
Survival analysis in glioma patients with different levels of LYN expression. Kaplan–Meier curve of OS in glioma samples in **(A)**. TCGA and **(B)**. CGGA. *p*-values were calculated based on log-rank test **(C)**. Univariate cox regression analysis evaluating prognostic value of LYN expression in different cancer types regarding OS in TCGA. The length of horizontal line represents the 95% confidence interval. The vertical dotted line represents the HR of cancer patients. HR < 1.0 indicates that high LYN expression is a favorable prognostic biomarker.

Next, the prognostic value of LYN expression was explored. Patients with high LYN expression exhibited significantly lower overall survival (OS) than patients with low LYN expression in TCGA and CGGA (Figures 2A,B). Moreover, LYN expression was negatively related to progression-free interval (PFI) and disease-specific survival (DSS) in glioma patients (Supplementary Figure S2). ROC curve further indicated that LYN was a sensitive marker for 3-years and 5-years survival (Supplementary Figure S1G). The prognostic value of LYN was further validated using other datasets including CGGA325, CGGA693, array300, and GSE108474 (Supplementary Figure S3). Although the survival difference in GBM samples from TCGA (*p* = 0.16), CGGAarray (*p* = 0.55), and GSE108474 (*p* = 0.65) was not statistically significant, this could be attributed to the insufficient samples volume in these three datasets as the tendency in survival difference was evident that high LYN expression could predict worse survival. In the pan-cancer analysis, LYN indicated worse OS in thymoma (THYM), uveal melanoma (UVM), uterine carcinosarcoma (UCS), acute myeloid leukemia (LAML), kidney renal papillary cell carcinoma (KIRP), uterine corpus endometrial carcinoma (UCEC), liver hepatocellular carcinoma (LIHC), mesothelioma (MESO), breast invasive carcinoma (BRCA), ovarian serous pancreatic adenocarcinoma (PAAD), and testicular germ cell tumors (TGCT) (Supplementary Figure S4). We further revealed that LYN expression was a hazardous marker in eleven cancer types and a favorable marker in seven cancer types (Figure 2C).

**FIGURE 3:**
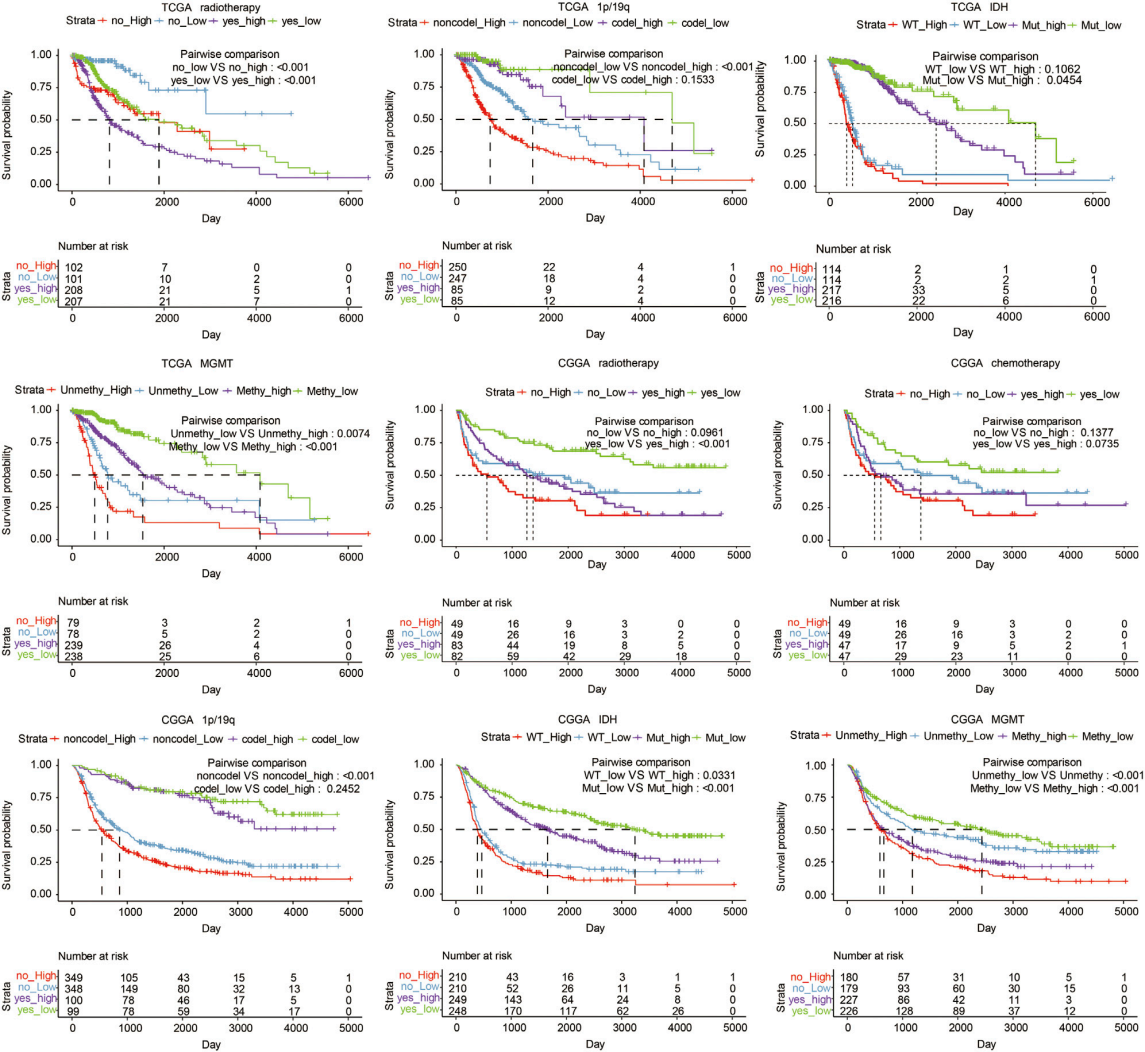
Overall survival in glioma patients with different status, including radiotherapy, chemotherapy, 1p19q, MGMT, and IDH in TCGA and CGGA datasets.

In TCGA, high LYN expression was associated with reduced survival in glioma patients regarding different IDH statuses (*p* = 0.0454, *p* = 0.1062, respectively; Figure 3), radiotherapy statuses (*p* < 0.001, respectively; Figure 3), 1p19q statuses (*p* < 0.001, *p* = 0.1533, respectively; Figure 3), and MGMT promotor statuses (*p* = 0.0074, *p* < 0.001, respectively; Figure 3). Likewise, in CGGA, high LYN expression predicted worse survival in glioma patients regarding different IDH statuses (*p* = 0.0331, *p* < 0.001, respectively; Figure 3), radiotherapy statuses (*p* = 0.0961, *p* < 0.001, respectively; Figure 3), chemotherapy statuses (*p* = 0.1377, *p* = 0.0735, respectively; Figure 3), 1p19q statuses (*p* < 0.001, *p* = 0.2452, respectively; Figure 3), and with MGMT promotor statuses (*p* < 0.001, respectively; Figure 3). Notably, glioma patients receiving radiotherapy experienced reduced survival in TCGA (*p* < 0.001, respectively; Figure 3), which suggested that LGG patients might negatively respond to radiotherapy.

### LYN Expression Correlates With Genomic Alterations

**FIGURE 4:**
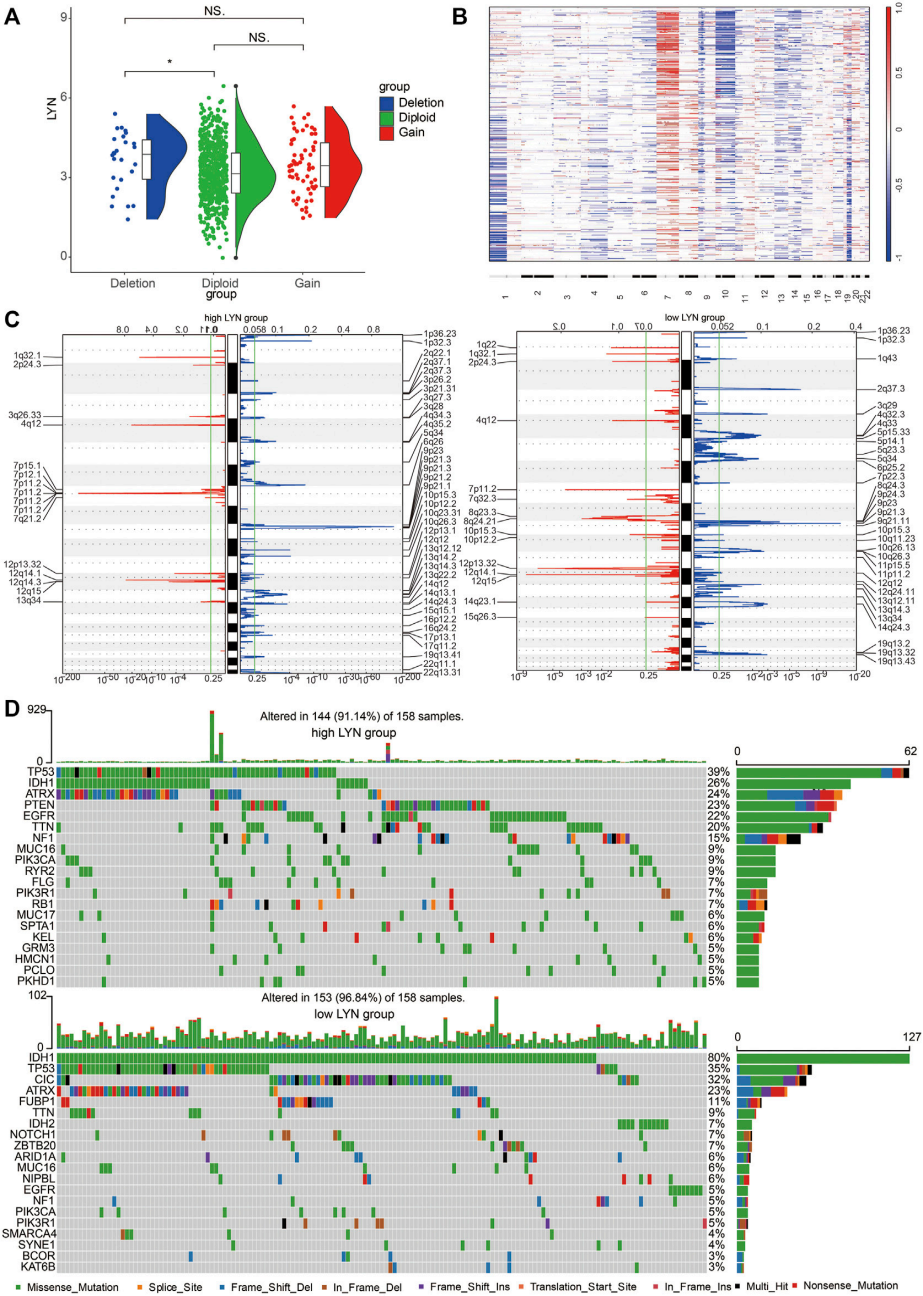
Different genomic alterations are related to LYN expression **(A)**. Relationship between LYN expression and copy number variation in TCGA. NS, *, **, and *** indicate *p* < 0.05, *p* < 0.01, *p* < 0.001, and no significant difference, respectively **(B)**. The overall CNA profile based on LYN expression. 22 human chromosomes are numbered 1 to 22 consecutively **(C)**. Genomic events in glioma samples with low and high LYN expression based on GISTIC 2.0. Chromosomal locations of amplification peaks (red) and deletions peaks (blue) are presented **(D)**. Different genomic alterations in glioma samples with low and high LYN expression.

Glioma samples with CN loss expressed significantly higher level of LYN mRNA than diploid (Figure 4A). A global CNA profile was obtained in glioma samples with low LYN expression and glioma samples with high LYN expression (Figures 4B,C). Glioma samples with high LYN expression had frequent amplification of chr7 and deletion of chr10 (Figure 4B), while glioma samples with low LYN expression had frequent deletion of 1p and 19q (Figure 4B). In glioma samples with high LYN expression, oncogenic driver genes such as EGFR (7p11.2) and CDK4 (12q14.1) were frequently amplified, while tumor suppressor gene PTEN (10q23.31) and CDKN2A (9p21.3) were frequently deleted (Figure 4C). Additionally, TP53 (39%), PTEN (23%), EGFR (22%), TTN (20%) were frequently mutated in the LYN high expression cluster, while IDH1 (80%) and CIC (32%) were frequently mutated in the LYN low expression cluster (Figure 4D). It should be noted that ATRX had similar mutation rate in LYN high expression cluster and LYN low expression cluster, which indicated that the mutation of ATRX might be independent of LYN expression.

### LYN Mediates Tumor Immune Microenvironment

**FIGURE 5:**
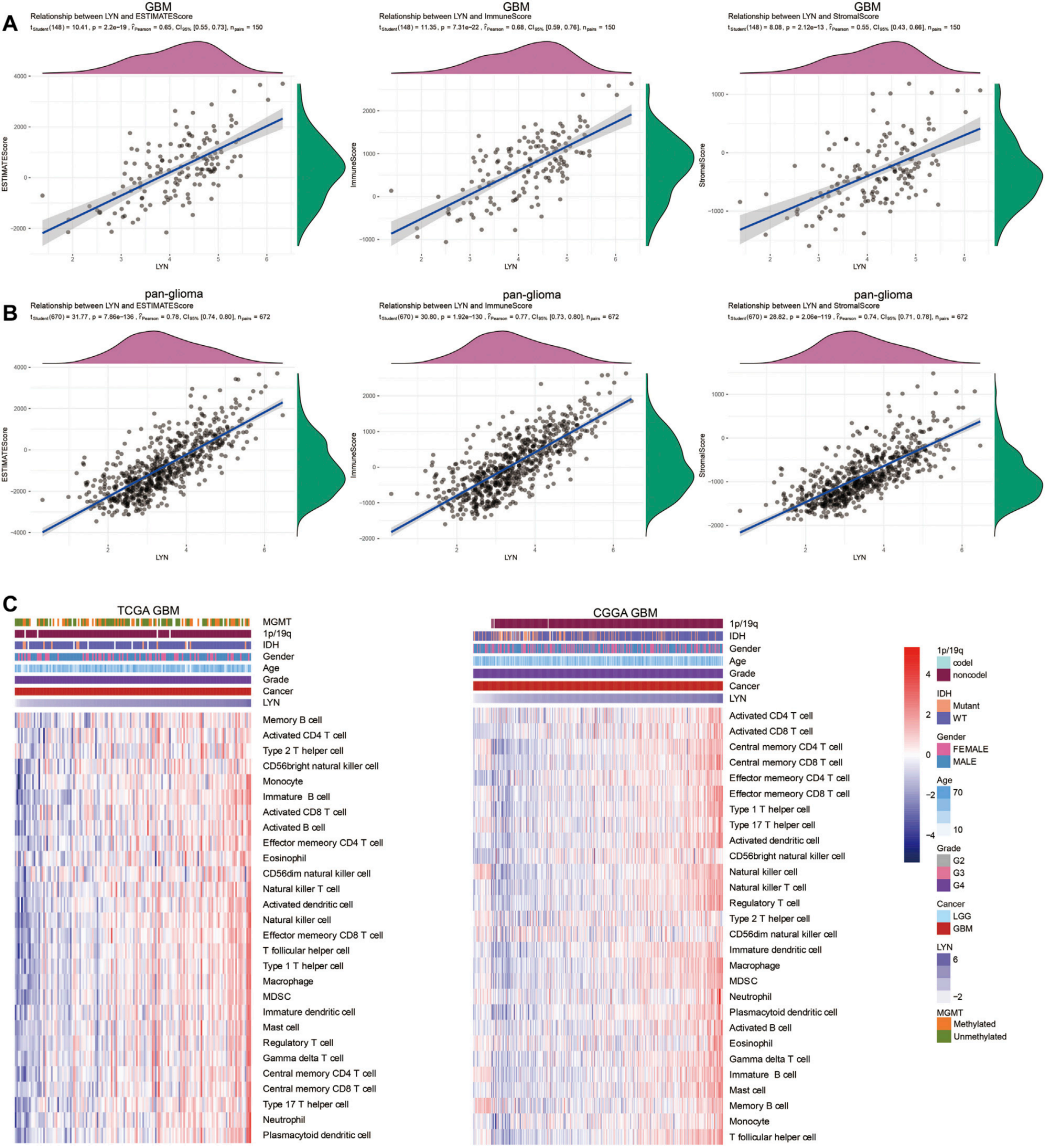
LYN correlates with immune infiltration **(A)**. The correlation between LYN and Estimate Score, Immune Score, and Stromal Score in GBM samples in TCGA **(B)**. The correlation between LYN and Estimate Score, Immune Score, and Stromal Score in pan-glioma samples in CGGA **(C)**. Heatmaps illustrating the relationship between LYN and 28 immune cell types in TCGA GBM samples and CGGA GBM samples, respectively. The expression values are z-transformed. High expression values are colored red and low expression values are colored blue.

**FIGURE 6:**
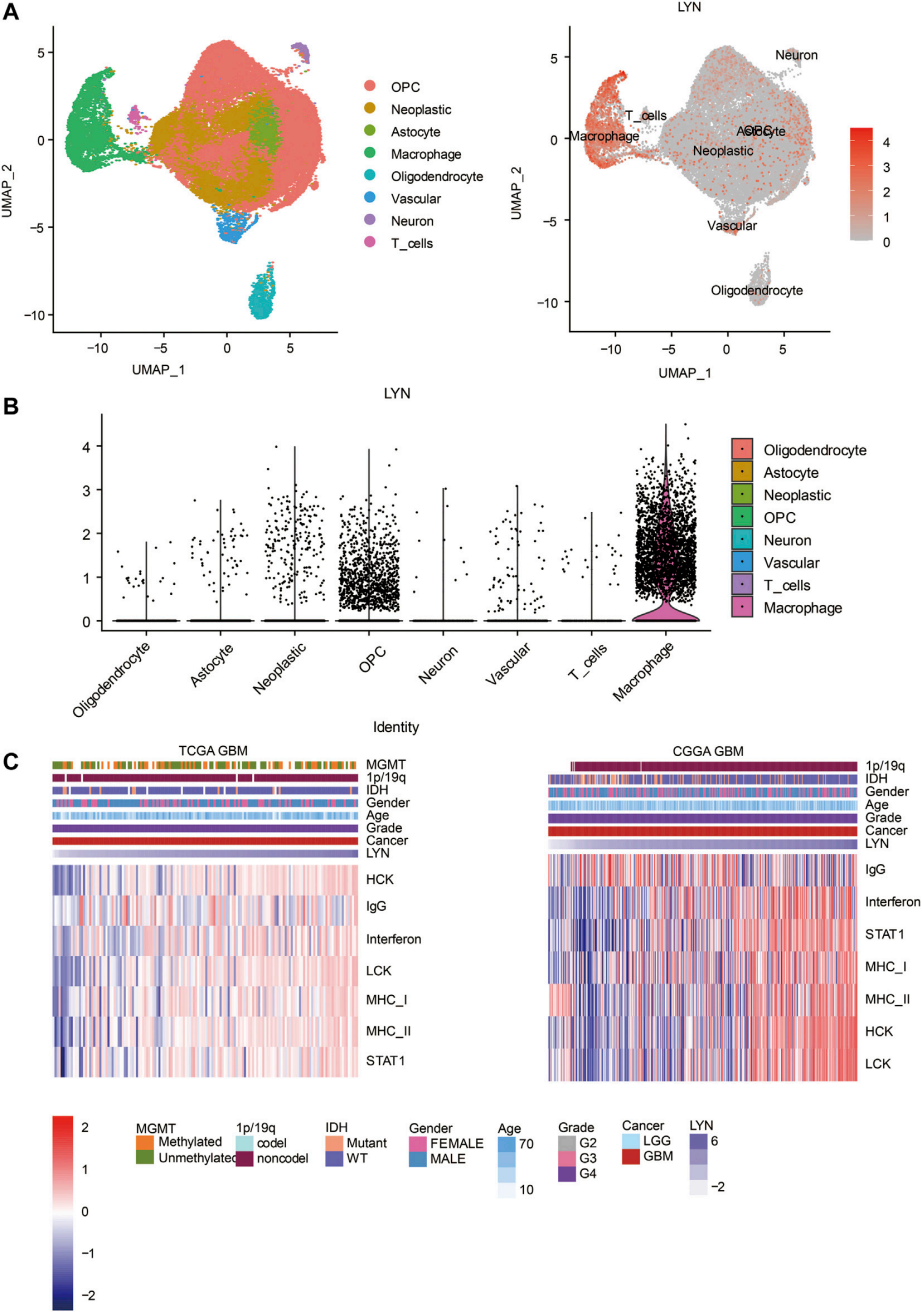
Single cell sequencing analysis **(A)**. UMAP plot showing 8 cell clusters. Gray area represents all clustered cells. The red dot represents cell expressing LYN **(B)**. The expression level of LYN in 8 cell clusters **(C)**. Heatmaps illustrating LYN related inflammatory activities in TCGA GBM and CGGA GBM, respectively. The expression values are z-transformed. High expression values are colored red and low expression values are colored blue.

We identified the relationship between LYN and ESTIMATE scores. LYN was found to positively correlate with immune score, stromal score, and ESTIMATE score in GBM samples (Figure 5A) and glioma samples (Figure 5B). LYN expression was positively associated with a variety of immune infiltrating cell types responsible for an anti-tumor response in GBM samples (Figures 5C,D) and glioma samples (Supplementary Figures S5A,B). Additionally, based on the CIBERSORT algorithm, high LYN expression positively correlated with M2 macrophages, neutrophils (Supplementary Figures S5C,D). In single-cell sequencing analysis of GBM samples, after regressing out the patient effects, eight clusters of cells were identified (Figure 6A). The expression pattern of LYN in the eight-cell types was shown in Figure 6A. Moreover, the relative expression level of LYN was shown in Figure 6B, which confirmed that LYN highly correlated with macrophage and oligodendrocyte precursor cell (OPC). This result suggested that the abnormal high expression of LYN in GBM could be potentially caused by immune infiltrating microenvironment dominated by macrophages. Besides, LYN expression correlated with inflammatory signature genes in glioma samples (Supplementary Figures S6A,B) and GBM samples (Figure 6C) in TCGA and CGGA.

### LYN Correlates With Other Immune Checkpoint Molecules and Immune-Related Pathways in Gliomas

**FIGURE 7:**
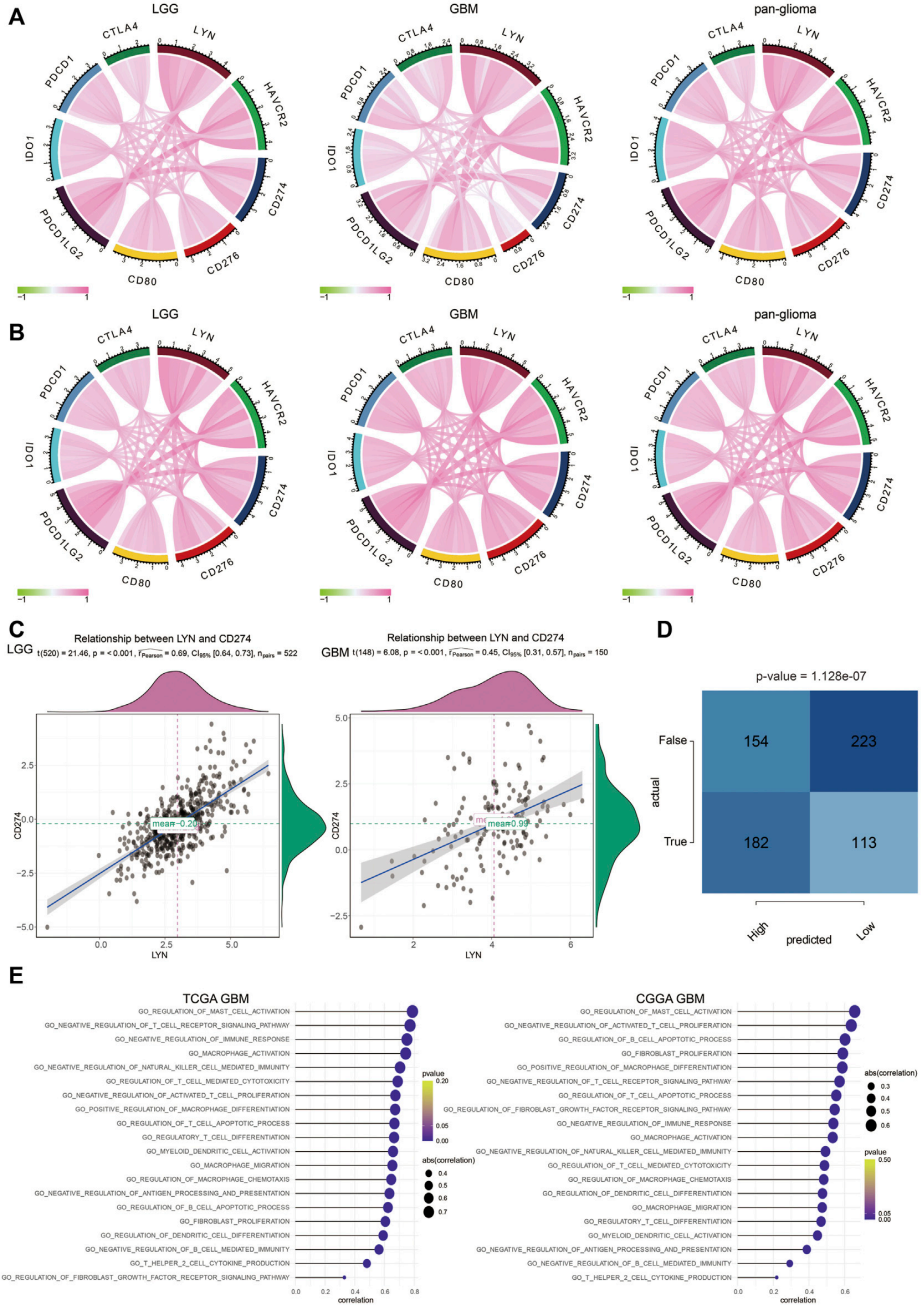
LYN is associated with immunosuppressive activities **(A)**. The correlation between LYN and classical immune checkpoints in TCGA dataset **(B)**. The correlation between LYN and classical immune checkpoints in CGGA dataset **(C)**. The correlation between LYN and CD274 (PD-L1) in LGG and GBM in TCGA **(D)**. Contingency table showing the predictive value of LYN expression in immunotherapy responses based on TIDE algorithm **(E)**. The correlation between LYN and immune related signaling pathways in TCGA GBM and CGGA GBM samples in GO analysis.

As shown in Figures 7A,B, LYN positively correlated with classical immune checkpoint molecules in glioma samples in TCGA and CGGA. The correlation between LYN and PD-L1 was further analyzed in LGG and GBM samples, and the correlation was found to be higher in LGG than GBM (Figure 7C). Moreover, LYN expression in glioma patients could significantly predict anti-PD-1 and anti-CTLA-4 immunotherapy responses based on the TIDE algorithm (Figure 7D). Given that, LYN was more conceivably involved in the process of modulating immunosuppression-related signaling pathways by combining with other immune checkpoints in the LGG microenvironment.

**FIGURE 8:**
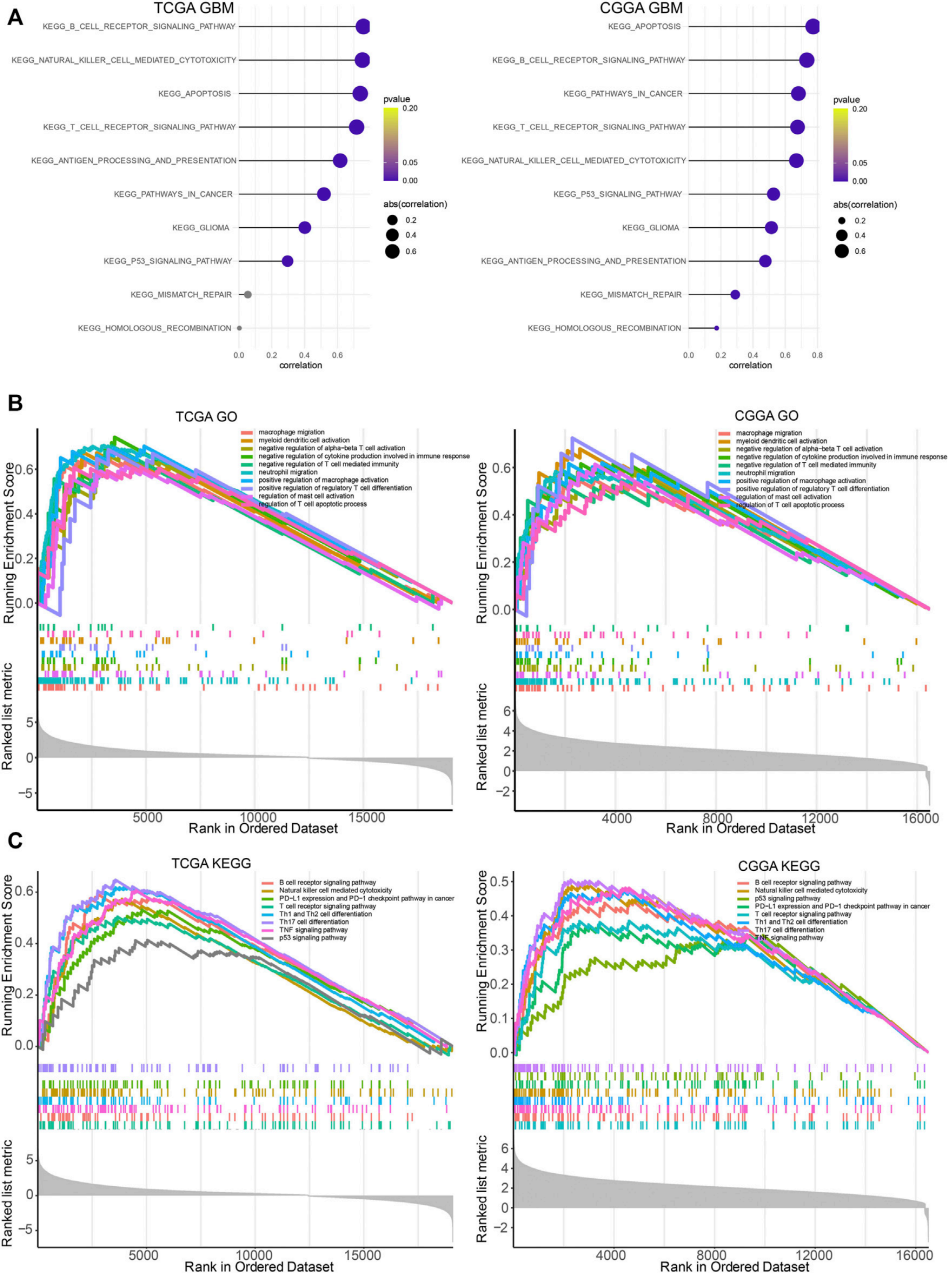
LYN related immune processes **(A)**. The correlation between LYN and immune related signaling pathways in TCGA GBM and CGGA GBM samples in KEGG analysis **(B)**. GSEA of LYN in TCGA and CGGA in GO **(C)**. GSEA of LYN in TCGA and CGGA in KEGG.

LYN was found to positively correlate with regulation of B cell immunity, T cell proliferation, regulatory T cell differentiation, T cell apoptotic process, CD4 positive alpha-beta T cell activation, and regulation of T cell differentiation in GBM samples (Figure 7E) and glioma samples (Supplementary Figures S7A,B) in TCGA and CGGA based on GSVA results of GO terms. Thus, these findings indicated that LYN critically regulated the tumor immune environment in gliomas. In GSVA results of KEGG terms, LYN was associated with apoptosis, pathways in cancer, p53 signaling pathway, and mismatch repair in GBM samples (Figure 8A) and glioma samples (Supplementary Figures S7C,D) in both datasets. The GSEA results in GO (Figure 8B) and KEGG (Figure 8C) further confirmed that LYN correlated with immune suppressive activity and tumor proliferation in TCGA and CGGA.

### Identification of Gene Modules Associated With LYN Expression

**FIGURE 9:**
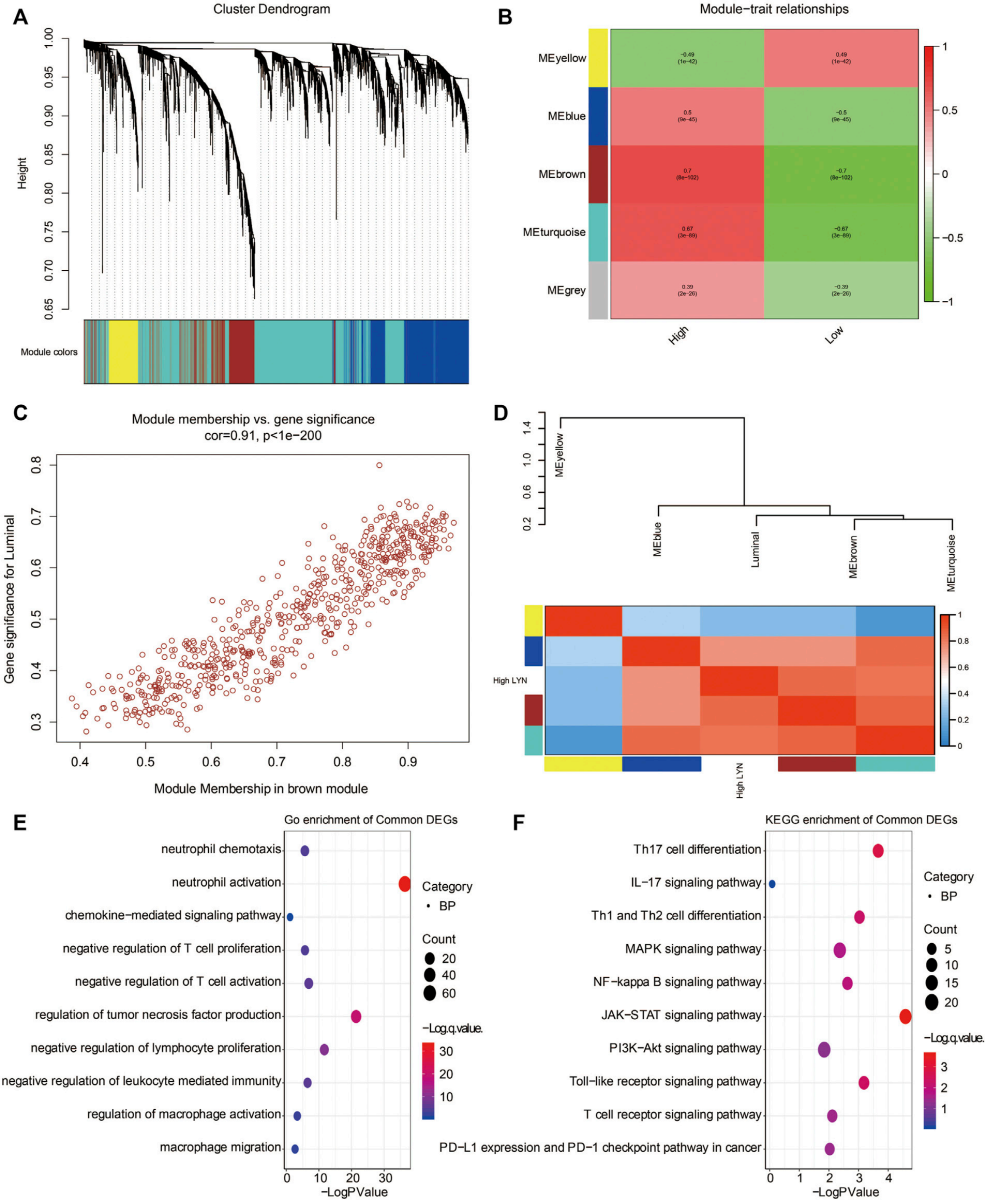
WGCNA exploring LYN-related gene module **(A)**. WGCNA was performed to identify five modules **(B)**. Four modules (nongrey) were identified with the brown module showing the highest correlation (*r* = 0.7, *P* = 8e-102) with high LYN expression **(C)**. The gene significance highly correlated with module membership of the genes in the brown module **(D)**. The clustering results of the identified modules **(E)**. GO enrichment analysis of the genes extracted from brown module **(F)**. KEGG enrichment analysis of the genes extracted from brown module.

WGCNA analysis was used to determine the most correlated function of LYN based on the cluster dendrogram of LYN-related genes (Figure 9A). Genes were grouped into five modules, and the correlation coefficient between the identified modules and the expression level of LYN was calculated (Figures 9B,D). Notably, the brown module exhibited the highest correlation coefficient with a high LYN expression level (Figure 9B, *r* = 0.7, *p* < 8e-102). A significant correlation for genes in the brown module is illustrated in the plots of module membership and gene significance (Figure 9C, *r* = 0.91, *p* < 1e-200). GO analysis revealed that neutrophil activation and chemotaxis, negative regulation of T cell proliferation and activation, and chemokine-mediated signaling pathways were the most related gene functions associated with the high expression of LYN (Figure 9E). KEGG analysis revealed that Th17 cell differentiation, IL-17 signaling pathway, Th1 and Th2 cell differentiation, as well as NF-kappa B signaling pathway, were the pathways most related to the high expression of LYN (Figure 9F).

## Discussion

Immunotherapy is a rising star in tumor treatment, and immune checkpoint blockade has demonstrated promising results. Given the increasing attention to incorporate ICB in the treatment of gliomas, exploring novel and potential immune checkpoint molecules are significant. Based on large-scale bioinformatics analysis, we comprehensively delineated the clinical characteristic landscape of LYN in the immune infiltrating microenvironment of gliomas. Our work revealed that LYN was upregulated in higher grade gliomas. LYN expression was also highly correlated with IDH wildtype in GBM and served as a sensitive marker of IDH status. Besides, LYN was enriched in CL and ME subtype of gliomas, which indicated worse survival, and LYN was associated with 1p19q non-codeletion and unmethylated MGMT promoter, both of which predicted worse survival in glioma patients. LYN was mostly localized in hyperplastic blood vessels region that created a permissive environment for tumor growth.

LYN has been reported to promote the proliferation and migration of glioma cells and inversely correlates with patient survival (Dhruv et al., 2014; Lewis-Tuffin et al., 2015; Moncayo et al., 2018). In this study, LYN was associated with worse survival in both LGG and GBM patients. Additionally, genomic alternations analysis revealed that LYN expression was positively associated with somatic mutations and CNVs. Amplification of oncogenic drivers including PDGFRA, EGFR, and CDK4 were detected in high LYN expression samples, while tumor suppressor genes including CDKN2A/CDKN2B and PTEN had a deletion peak in high LYN expression samples. However, only in*in vivo* and *in vitro* experiments with LYN being knocked down or overexpressed in cells or animals, could we prove that these high frequency mutations are closely related to LYN. The abnormal higher mutation rate of tumor suppressor gene, TP53, in LYN high expression cluster was intriguing and also needed to be explored. Taken together, these results indicated that LYN regulated glioma cell progression and proliferation, and high LYN expression could predict the survival rate of glioma patients.

Previous studies have demonstrated that LYN regulates the development and function of multiple immune cells, such as macrophage, dendritic cells (Yang et al., 2018), T cells (Fallacara et al., 2019), and B cells (Brodie et al., 2018). This study further showed that LYN was involved in the glioma immune microenvironment. Besides, LYN was found to have a positive correlation with estimate score, immune score and stromal score, all of which were negatively correlated with the prognosis of glioma patients (Jia et al., 2018). LYN also positively correlated with various immune infiltrating cells, including Tregs, M2 macrophage, and MDSC, which contribute to an immunosuppressive microenvironment in gliomas (Marvel and Gabrilovich, 2015; Zhang et al., 2021d; Zhang et al., 2021e; Zhang et al., 2021f). GSVA in GO confirmed that LYN was involved in macrophage activation, macrophage migration, fibroblast proliferation, myeloid dendritic cell activation, regulatory T cell differentiation, and negative regulation of activated T cell proliferation. Additionally, GSVA in KEGG and GSEA showed that LYN negatively regulated the immune system in gliomas and promoted the proliferation of glioma cells. Besides, a correlation was observed between inflammatory signatures and LYN. Taken together, these results revealed the immuno-suppressive role played by LYN in gliomas.

Immune checkpoint blockage enhances anti-tumor immune response. Several classical immune checkpoint molecules including PD-1, PD-L1, LAG3, and TIM3, have been shown to cause disorders of the immune system (Li et al., 2018). The classical PD-1/PD-L1 axis directly promotes the invasion and progression of GBM cells (Litak et al., 2019). In this study, LYN remarkably correlated with multiple classical immune checkpoint molecules. Special attention was paid to the connection between LYN and PD-L1, and LYN showed a correlation with PD-L1 in LGG and GBM. These results showed that LYN could potentially mediate the function of immune checkpoint molecules.

Taken together, we described the characteristics of LYN in gliomas based on bioinformatics analysis of several datasets. LYN predicted malignant gliomas and served as a prognostic marker indicating worse survival of glioma patients. Moreover, LYN facilitated the establishment of an immune-suppressive and favorable glioma microenvironment. However, there is a need for research to investigate LYN as a potential immune checkpoint molecule, which can promote the clinical management of glioma patients receiving immunotherapy.

## Materials and Methods

### Data Collection

The transcriptome data of LYN was collected from glioma samples in TCGA and CGGA databases. 672 glioma samples were downloaded from TCGA (https://xenabrowser.net/). Three CGGA cohorts, including mRNAseq_693 (693 glioma samples), mRNAseq_325 (325 glioma samples), and CGGAarray (300 glioma samples), were downloaded from CGGA (http://www.cgga.org.cn/) and included in this study. CGGA325 and CGGA693 were combined as CGGA dataset after removing batch effect using the R package sva. 414 glioma samples from GSE108474 dataset were downloaded from the GEO database (https://www.ncbi.nlm.nih.gov/geo/). Fragments per kilobase million (FPKM) values of gene matrix were transformed into transcripts per kilobase million (TPM) values. RNA-seq data of tumor localization in patients with GBM was downloaded from the Ivy Glioblastoma Atlas Project (http://glioblastoma. alleninstitute.org/). A total of 8,295 normal samples were collected from GTEX (http://commonfund.nih.gov/GTEx/) and TCGA databases. Single-cell sequencing dataset was downloaded from GSE138794 (Wang et al., 2019) in the Gene Expression Omnibus (GEO; https://www.ncbi.nlm.nih.gov/) database. Eight scRNA sequencing samples of GBM were included in the analysis. Tumor tissues were from glioma patients underwent surgery in Xiangya Hospital, Central South University. Written informed consent was obtained from all patients.

### Immunohistochemistry

Paraffin-embedded tissue sections were obtained from human gliomas (WHO grades II-IV) and used for IHC staining. 40 samples were used for LYN. The sections were incubated with the LYN primary antibody (Rabbit) (1:50; Proteintech; Wuhan, China) after blockage with 5% BSA. The antibody reaction was visualized after 3, 3′-diaminobenzidine (DAB) development, and sections were counterstained with hematoxylin.

### Biological Function Annotation of LYN

Somatic mutations and copy number alterations (CNAs) of glioma samples were downloaded from TCGA. GISTIC 2.0 (https://gatkforums.broadinstitute.org) was performed for enrichment of genetic alteration events. CIBERSORT algorithm was performed for quantifying the expression level of 22 immune cell types (Newman et al., 2015). The gene sets variation analysis (GSVA) and gene set enrichment analysis (GSEA) were applied to investigate the immune-related and tumor-related processes of LYN. ssGSEA was used for the enrichment pattern of the 28 immune cell types. TIDE algorithm was applied to evaluate the predictive value of LYN in anti-PD-1 and anti-CTLA-4 immunotherapy responses (Jiang et al., 2018).

### Single Cell RNA-Sequencing

Single-cell expression matrix was processed using R package Seurat V3.1.2. Expression data was normalized by “NormalizeData”, and 2000 highly variable genes (HVGs) was identified after performing “FindVariableGenes”. “FindIntegrationAnchors” and “Integratedata” was applied to merge eight GBM samples of single-cell sequencing (Stuart et al., 2019). Principal component analysis (PCA) was performed as previously described. Finally, “UMAP” was adopted for visualization of cell clusters with patient effects regressed out.

### Weighted Gene Co-expression Network Analysis

R package WGCNA was applied to explore the LYN related genes. After performing the correlation analysis between LYN and the gene expression matrix in TCGA, 3923 LYN related genes (correlation efficient >0.4) were used as the input of WGCNA. A power of β = 8 and a scale-free R2 = 0.84 were determined and set as soft-threshold parameters for a scale-free topology network. The calculated scale-free distribution topological matrix determined the softConnectivity for constructing sample dendrogram. The labeledHeatmap was used for depicting the module-trait relationship. The internal gene significance of each module was calculated and visualized using verboseScatterplot. Genes within the identified module with the highest gene significance were extracted for GO and KEGG enrichment analysis.

### Statistical Analysis

The datasets were divided into high and low groups based on the median and cutoff expression level of LYN in pan-gliomas, LGG, and GBM, respectively. Kaplan-Meier survival curves were generated using R package survival. The oneway analysis of variance was used to determine the LYN expression levels among multiple groups. The Wilcoxon rank testing was applied for determining the LYN expression levels in relation to different pathological characteristics between two groups. R package pROC was used for generation of receiver operating characteristic (ROC) curves. The classifier of ROC curve was built based on IDH status (WT vs. mutant) and molecular subtypes (classical, mesenchymal vs. proneural, neural). Pearson’s correlation coefficient was used for calculation of correlation coefficient. R version 3.6.3 was used for all statistical analyses. *p*-values < 0.05 were set as the criteria of statistically significant.

## Conclusion

LYN was associated with malignancy of gliomas and served as a prognostic marker of glioma patients. Besides, LYN facilitated the establishment of an immune-suppressive and favorable glioma microenvironment. At single cell sequencing level, LYN was abundantly expressed by tumor associated macrophages and T cells. Furthermore, the robust relationship between LYN and PD-L1 indicated that LYN might be a potent immune checkpoint molecule in predicting immunotherapy response.

## Data Availability

The original contributions presented in the study are included in the article/Supplementary Material, further inquiries can be directed to the corresponding authors.

## Abbreviations

CGGA, Chinese Glioma Genome Atlas; FPKM, Fragments per kilobase million; GBM, glioblastoma multiforme; GSVA, gene sets variation analysis; ICIs, immune checkpoints inhibitors; LGG, lower-grade glioma; LYN, Lck/yes-related protein tyrosine kinase; OS, overall survival; PD-1, programmed death 1; PD-L1, programmed death ligand 1; ROC, Receiver operating characteristic; TCGA, The Cancer Genome Atlas; TPM, transcripts per kilobase million; WHO, world health organization.
