# Supplementary material for: Immune Characteristics of LYN in Tumor Microenvironment of Gliomas
Source: Front Cell Dev Biol. 2022 Feb 2;9:760929. doi: 10.3389/fcell.2021.760929 (PMC8847791; doi:10.3389/fcell.2021.760929)
Supplement: Supplementary file 2 [file DataSheet1.docx]

Supplementary Figures


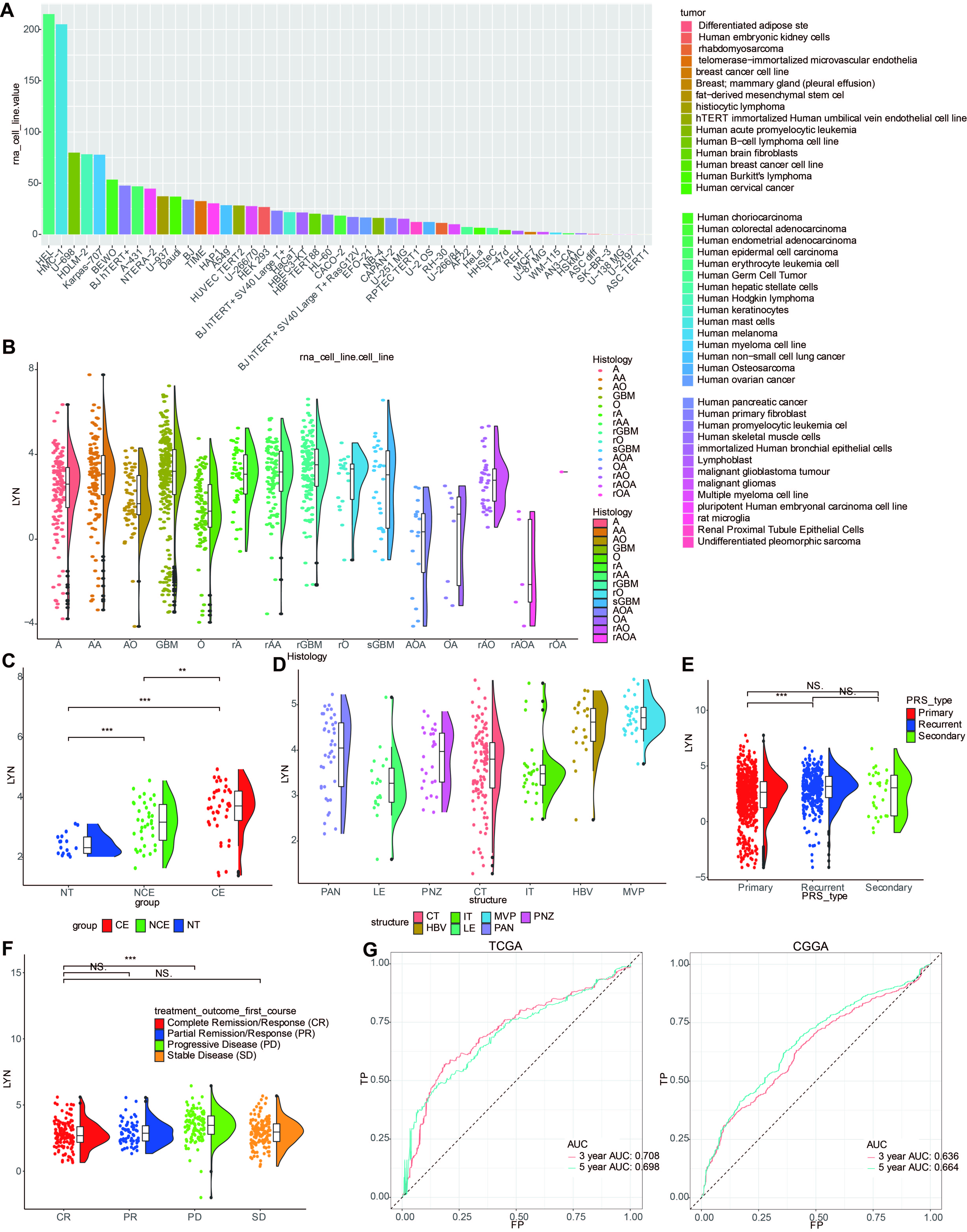


Figure S1. A. LYN expression in different tumor cell lines based on HPA database. B. LYN expression in different histopathologic classification from CGGA dataset. A, low-grade astrocytoma; AA, anaplastic astrocytoma; AO, anaplastic oligodendroglioma; GBM, glioblastoma; O, oligodendroglioma; rA, recurrent astrocytoma; rAA, recurrent anaplastic astrocytoma; rGBM, recurrent glioblastoma; rO, recurrent oligodendroglioma; sGBM, secondary glioblastoma; AOA, anaplastic oligoastrocytoma; OA, oligoastrocytoma. C. The LYN expression pattern in different radiographical regions in GILL dataset (GSE59612). Contrast-enhanced (CE), Non-contrast-enhanced (NCE), Normal tissues (NT). D. Intra-tumor analysis of LYN expression. LE (Leading Edge), IT (Infiltrating Tumour), CT (Cellular Tumour), PAN (Pseudopalisading Cells Around Necrosis), PNZ (Perinecrotic Zone), MVP (Microvascular Proliferation), and HBV (Hyperplastic Blood Vessels). E. LYN expression in primary, recurrent, and secondary gliomas. F. LYN expression in different treatment outcomes. G. ROC curves showed LYN as a predictor of 3-year and 5-year survival in TCGA and CGGA datasets.


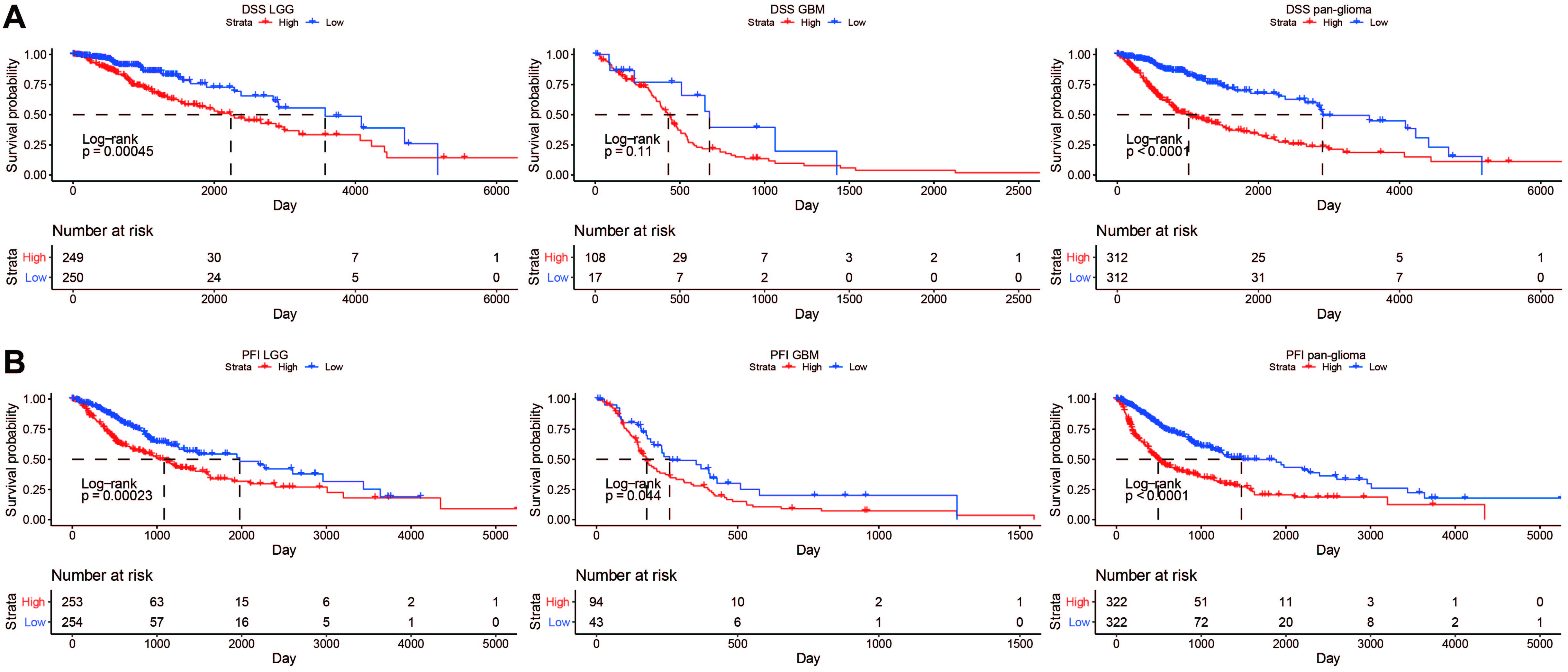


Figure S2. Disease specific survival and progressive free interval in glioma patients with low and high LYN expression. A. Kaplan – Meier analysis of disease specific survival was performed in pan-glioma, LGG and GBM patients. B. Kaplan – Meier analysis of progressive free interval was performed in pan-glioma, LGG and GBM patients.


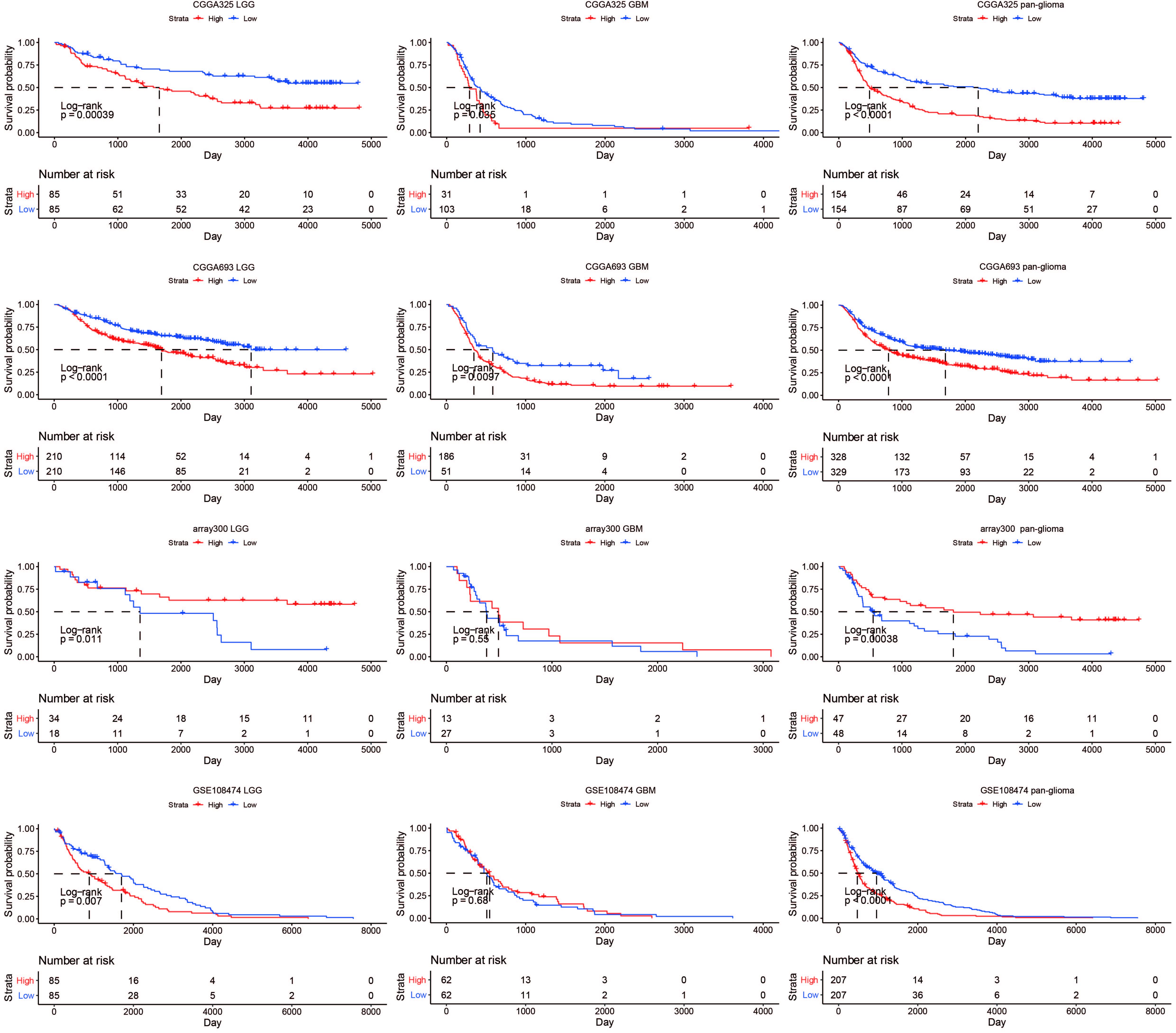


Figure S3. Overall survival in glioma patients with low and high LYN expression. Kaplan – Meier analysis of overall survival was performed in pan-glioma, LGG and GBM patients based on CGGA325, CGGA693, CGGAarray, and GSE108474 datasets.


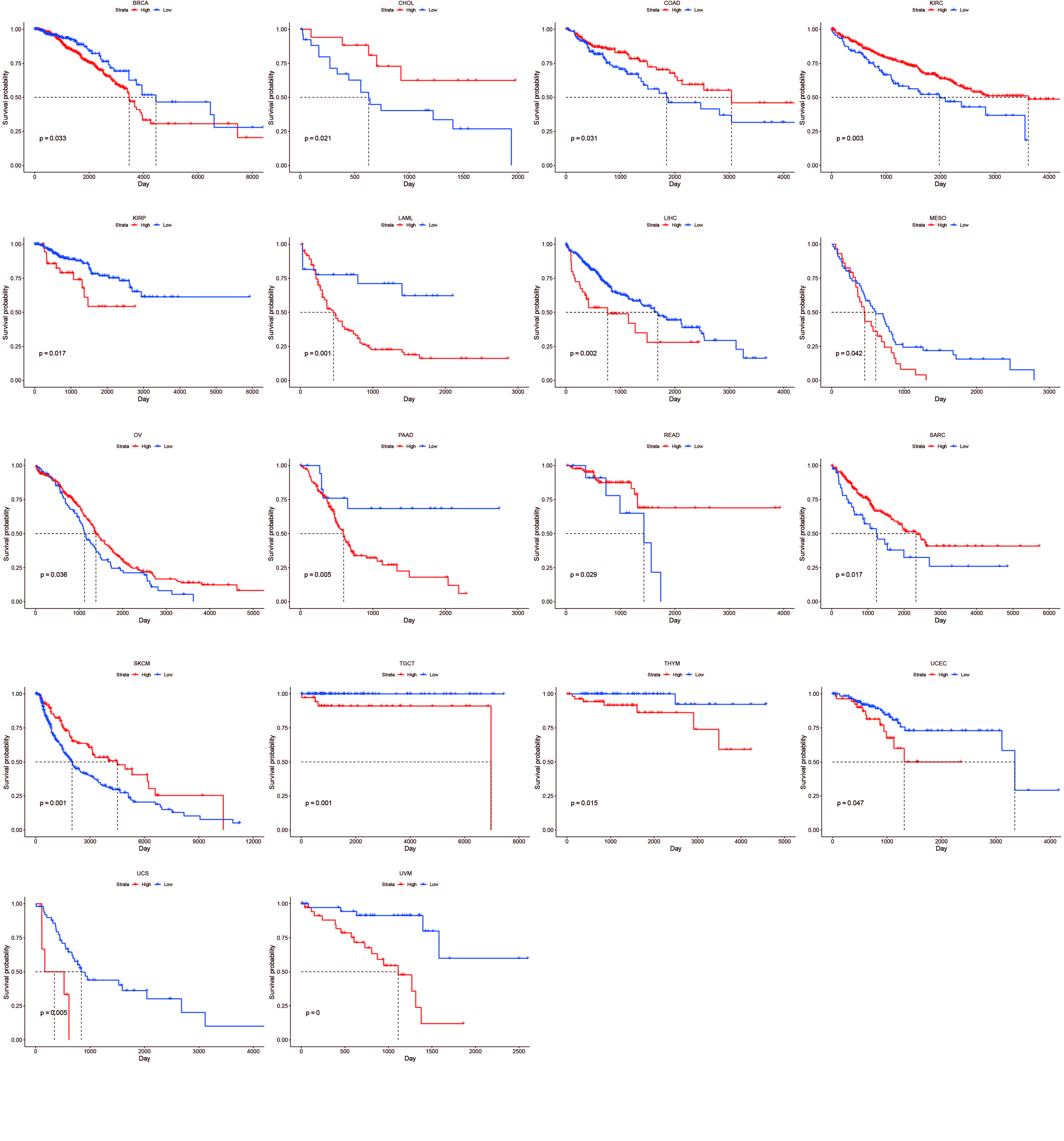


Figure S4. Overall survival in pan-cancer analysis. Kaplan – Meier analysis of overall survival was performed based on low and high LYN expression. BRCA, Breast invasive carcinoma; CHOL, Cholangiocarcinoma; COAD, Colon adenocarcinoma; KIRC, kidney renal clear cell carcinoma; KIRP, Kidney renal papillary cell carcinoma; LAML, Acute Myeloid Leukemia; LIHC, hepatocellular carcinoma; MESO, Mesothelioma; OV, Ovarian serous cystadenocarcinoma; PAAD, Pancreatic adenocarcinoma; READ, Rectum adenocarcinoma; SARC, Sarcoma; SKCM, Skin Cutaneous Melanoma; TGCT, testicular germ cell tumor; THYM, Thymoma; UCEC, Uterine Corpus Endometrial Carcinoma; UCS, Uterine Carcinosarcoma; UVM, Uveal Melanoma.


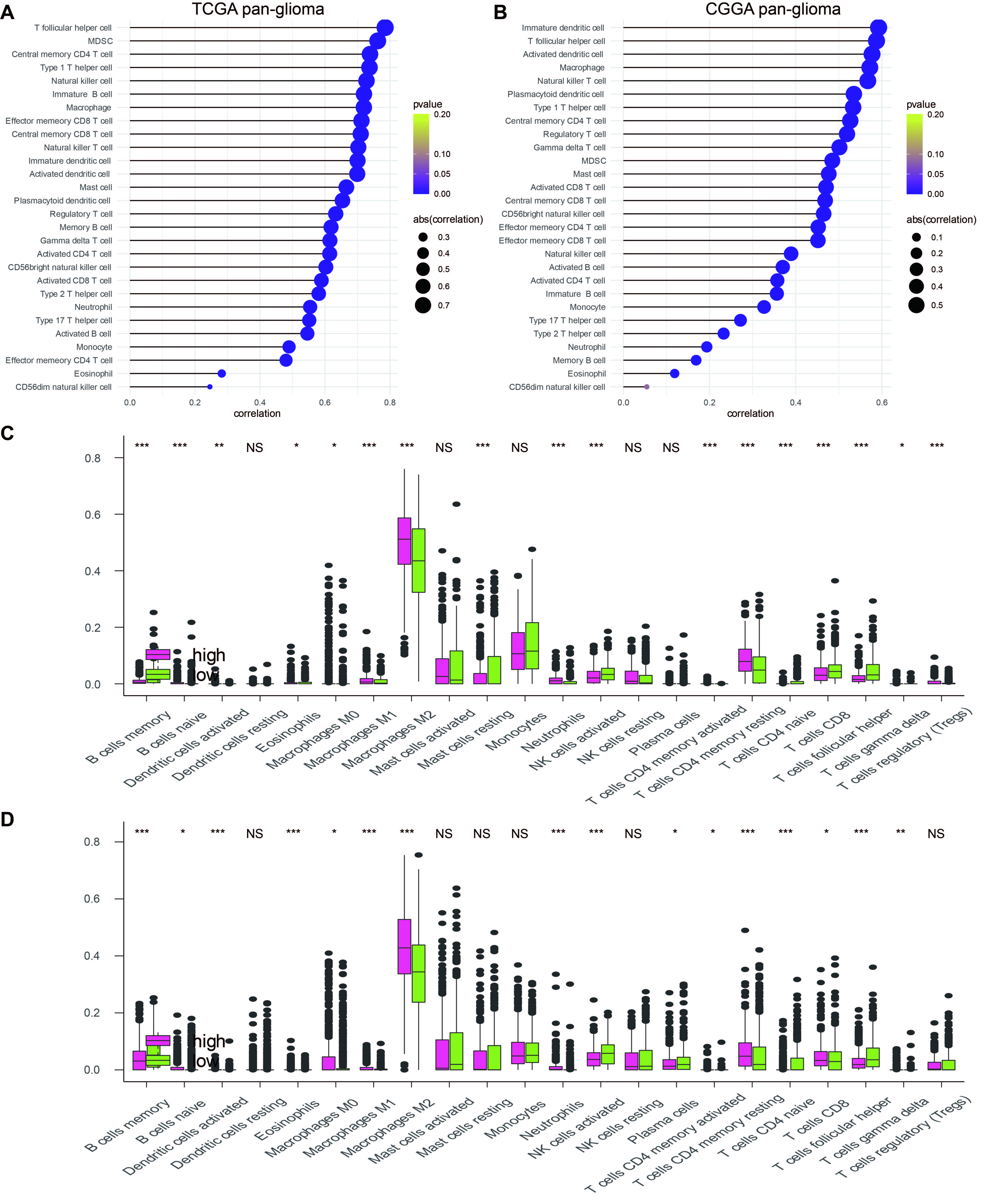


Figure S5. The relationship between LYN and 28 immune cell populations based on A. TCGA pan-gliomas and B. CGGA pan-gliomas, respectively. C. The fraction of 22 immune infiltrating cells in LYN in TCGA. Within each group, the scattered dots represent immune cell expression values. D. The fraction of 22 immune infiltrating cells in LYN in CGGA. Within each group, the scattered dots represent immune cell expression values.


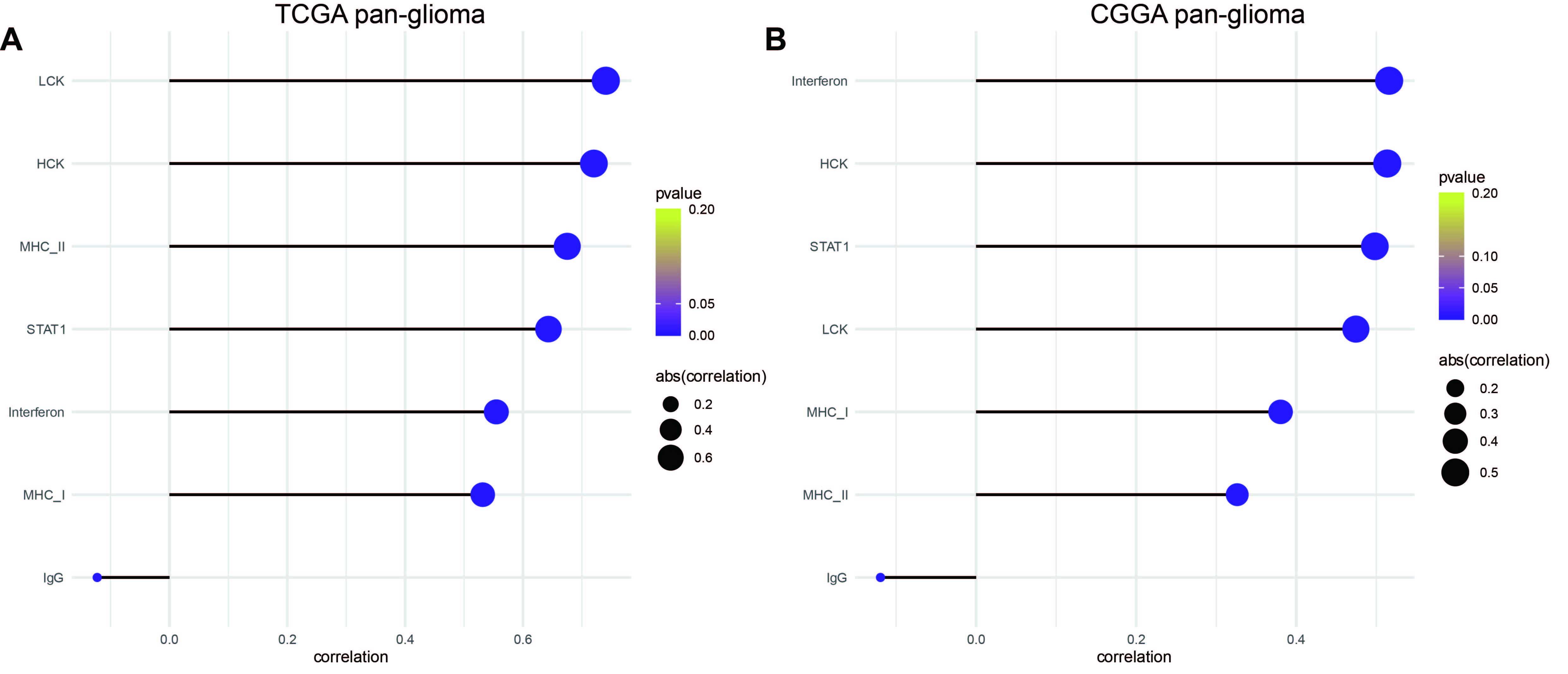


Figure S6. The relationship between LYN and inflammatory activities based on TCGA pan-gliomas and CGGA pan-gliomas, respectively.


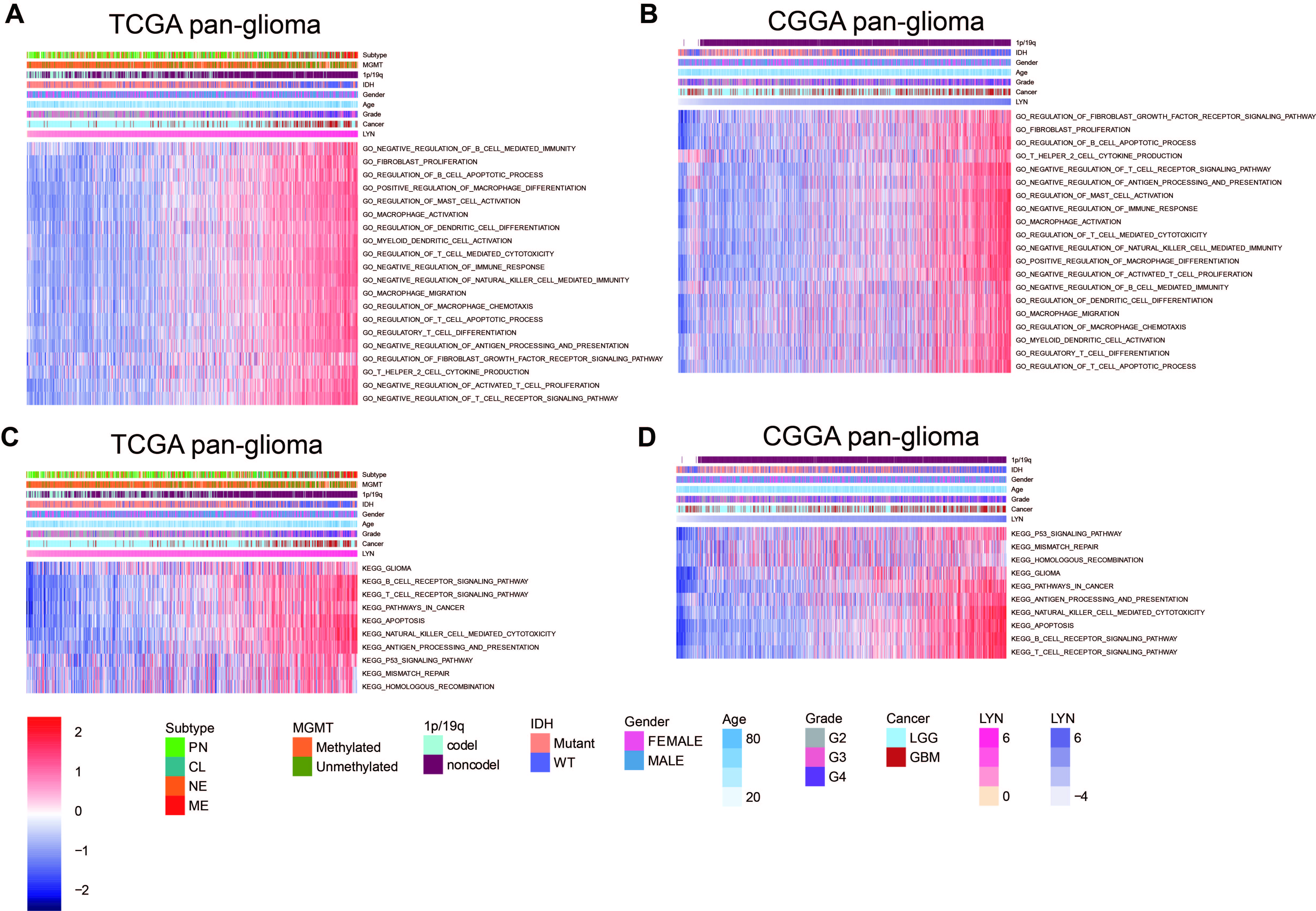


Figure S7. Heatmaps illustrating LYN related GO signaling pathways in A. TCGA pan-gliomas and B. CGGA pan-gliomas, respectively. Heatmaps illustrating LYN related KEGG signaling pathways in C. TCGA pan-gliomas and D. CGGA pan-gliomas, respectively. Expression values are z-transformed and are colored red for high expression and blue for low expression, as illustrated in the scale bar.

Table S1. Clinical characteristics of Xiangya cohort.
